# Orthodontic findings and treatment need in patients with amelogenesis imperfecta: a descriptive analysis

**DOI:** 10.1186/s13005-024-00436-y

**Published:** 2024-06-14

**Authors:** Stephan Christian Möhlhenrich, Sachin Chhatwani, Peter Schmidt, Kristian Kniha, Jan Postberg, Andreas G. Schulte, Jochen Jackowski, Stefan Zimmer, Gholamreza Danesh

**Affiliations:** 1 Department of Orthodontics, Witten/Herdecke University, Alfred-Herrhausen Str. 45, 58455 Witten, Germany; 2 Department of Special Care Dentistry, Witten/Herdecke University, Alfred-Herrhausen Str. 45, 58455 Witten, Germany; 3 Department of Oral and Maxillofacial Surgery, University Hospital of Aachen, Pauwelsstraße 30, 52074 Aachen, Germany; 4 Clinical Molecular Genetics and Epigenetics, Faculty of Health, Centre for Biomedical Education and Research (ZBAF), Witten/Herdecke University, Alfred-Herrhausen Str. 50, 58455 Witten, Germany; 5 Department of Oral Surgery, Witten/Herdecke University, Alfred-Herrhausen Str. 45, 58455 Witten, Germany; 6 Faculty of Health, Department of Dental Medicine, Department of Operative and Preventive Dentistry, Witten/Herdecke University, Alfred-Herrhausen-Str. 50, 58448 Witten, Germany

## Abstract

**Introduction:**

: Amelogenesis imperfecta (AI) is a genetically determined, non-syndromic enamel dysplasia that may manifest as hypoplasia, hypomaturation, or hypocalcification and can commonly be classified into four primary groups. In this retrospective analysis, specific orofacial characteristics are described and associated with each of the AI types based on a patient cohort from Witten/Herdecke University, Germany.

**Methods:**

Data from 19 patients (ten male and nine female, mean age 12.27 ± 4.06 years) with AI who presented at the Department of Orthodontics between July 2011 and December 2023 were analyzed. Baseline skeletal and dental conditions were assessed, including the presence of hypodontia, displacements, and taurodontism. AI was classified into classes I–IV based on phenotype. Treatment needs were evaluated according to the main findings following the German KIG classification, while the radiological enamel situation was determined using panoramic radiographs.

**Results:**

An approximately equal distribution between classes II and III was found and a slight inclination toward a dolichofacial configuration (ΔML-NSL: 5.07 ± 9.23°, ΔML-NL: 4.24 ± 8.04°). Regarding orthodontic findings, disturbance in tooth eruption as well as open bite were the most prevalent issues (both 36.8%, *n* = 7). The most common AI classes were type I and II, which show an almost even distribution about the skeletal classes in sagittal dimension, while dolichofacial configuration was found most frequently in vertical dimension.

**Conclusion:**

Both clinical and radiological orthodontic findings in context with AI are subject to extensive distribution. It seems that no specific orofacial findings can be confirmed in association with AI with regard to the common simple classes I–IV. It may be more appropriate to differentiate the many subtypes according to their genetic aspects to identify possible associated orthodontic findings.

## Introduction

Amelogenesis is a highly specialized, genetically controlled process that can be disrupted in various ways [1], resulting in enamel defects of varying severity. While isolated Amelogenesis Imperfecta (AI) is primarily a genetically determined, non-syndromic enamel dysplasia that may manifest as hypoplasia, hypomaturation, or hypocalcification [2, 3], it’s increasingly recognized that AI can also occur in syndromic forms [4], often alongside systemic conditions like nephrocalcinosis [5, 6]. This highlights the importance of considering systemic associations in AI patients. Dentists, as frontline healthcare providers, may contribute to early detection of renal abnormalities such as nephrocalcinosis through thorough patient assessments. Therefore, it’s crucial to address these systemic links when discussing AI presentations.

Among the known causal factors are mutations in genes exclusively responsible for encoding enamel proteins [7]. These genes encode enamel proteins including both structural (amelogenin [AMELX], enamelin [ENAM], ameloblastin [AMBN], odontogenesis associated phosphoprotein [ODAPH] and enzymatic (kallikrein 4 [KLK4], matrix metallopeptidase 20 [MMP20]) types. Others candidate genes encode transcription factors that regulate anatomical development (msh homeobox 2 [MSX2], distal-less homeobox 3 [DLX3]), a αvβ6 integrin receptor subunit (integrin subunit beta 6 [ITGB6]), and an cation carrier (solute carrier family 24 member 4 [SLC24A4]) as well as other proteins (WD repeat domain 72 [WDR72], family with sequence similarity 83 member A [FAM83H], collagen type XVII alpha 1 chain [COL17A1]) [8]. However, a review of current literature, with reported gene panel diagnostic rates ranging between 39% and 60%, suggests that for a significant proportion of all patients, the identified mutations alone are not causative of their AI symptoms [9, 10]. Consequently, diagnosing AI, even with the inclusion of molecular genetic investigations, remains associated with uncertainties.

**Fig. 1:**
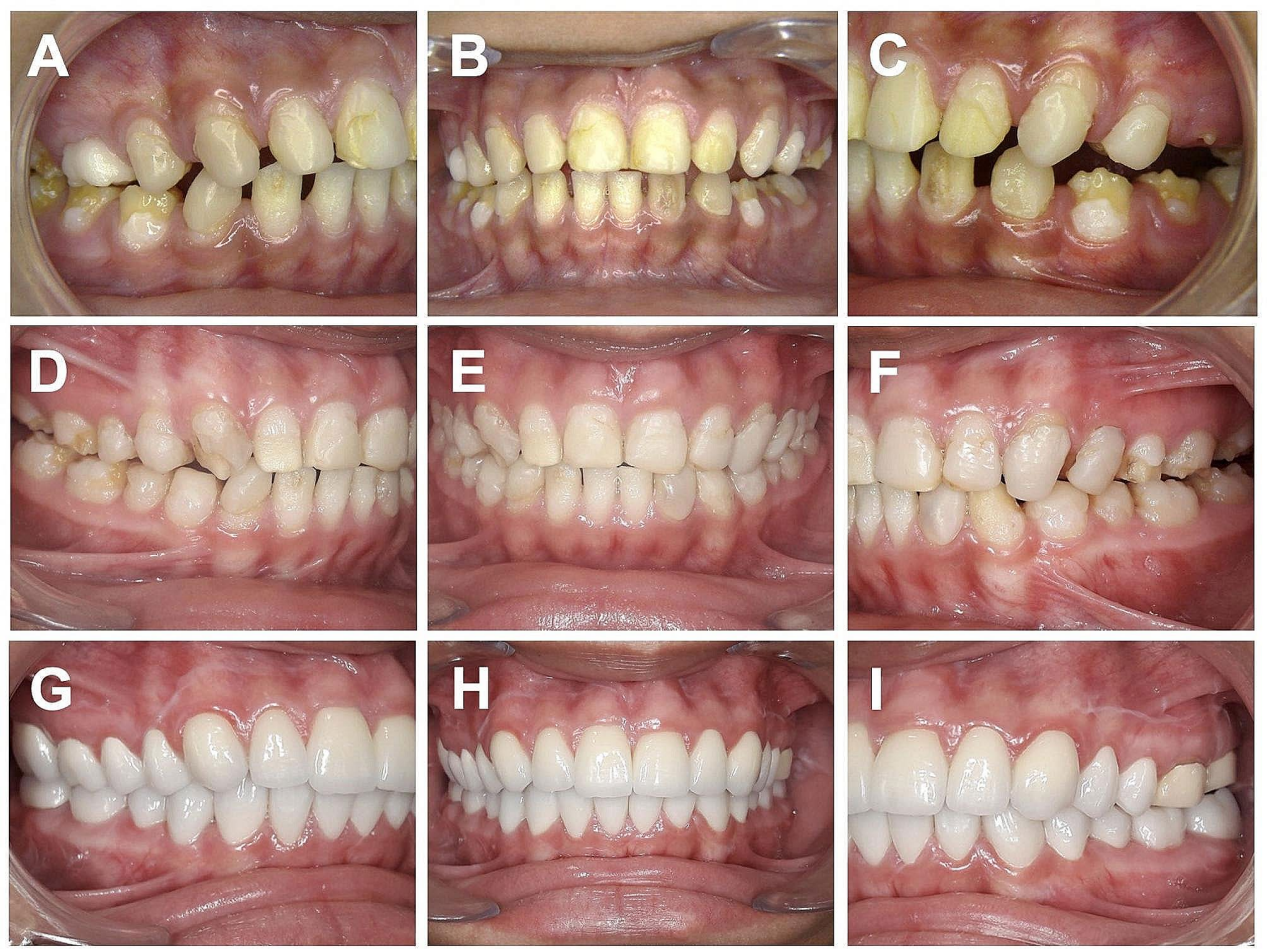
Interdisciplinary course of treatment of a patient with AI type I (hypoplastic). **A**–**C**) Initial findings with insufficient composite restorations of the entire permanent dentition. **D**–**F**) Intermediate findings after orthodontic treatment for generalized gap closure and midline correction. **G**–**I**) Final findings of prosthetic crown restoration

**Fig. 2:**
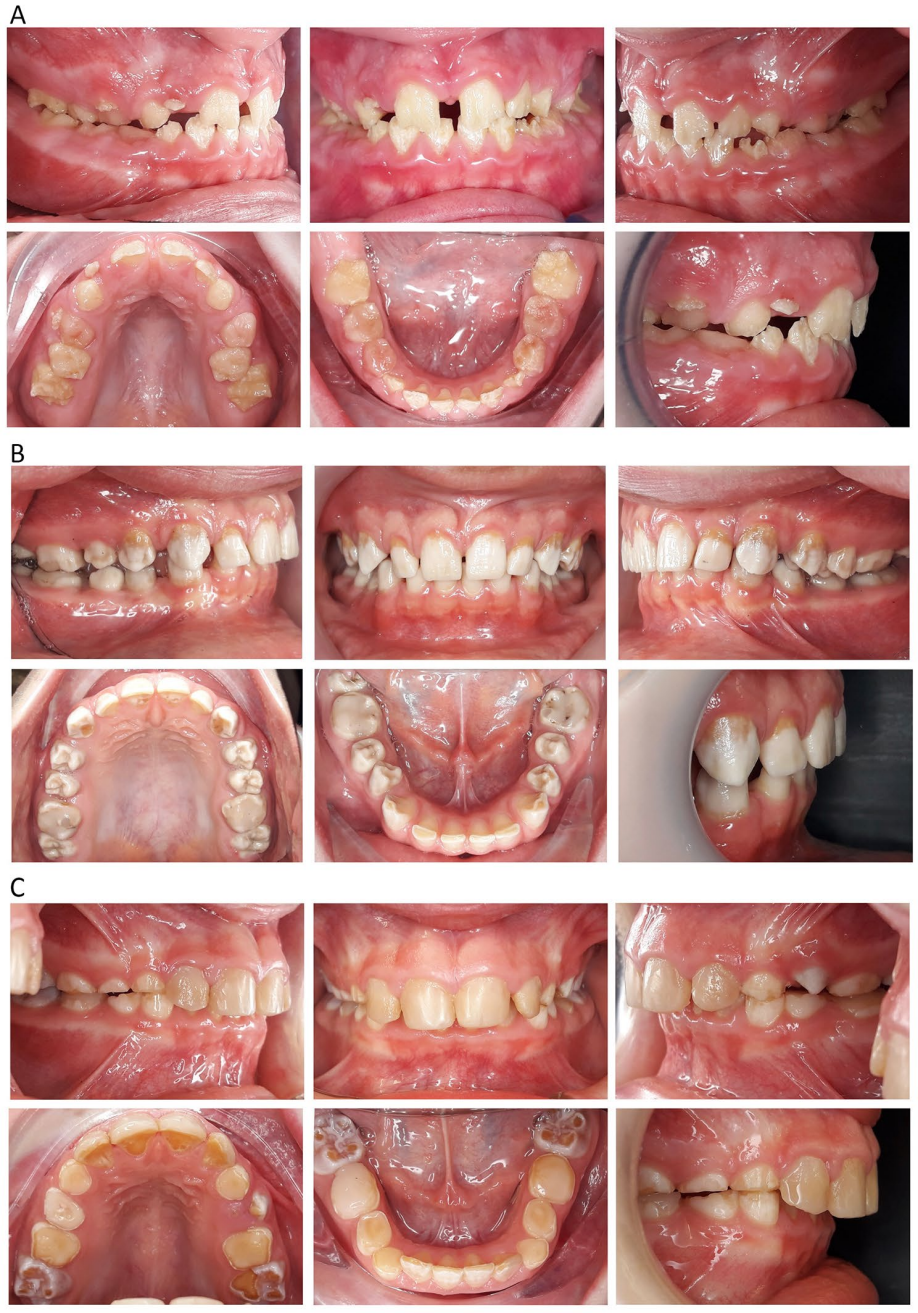
Descriptions of the phenotypic characteristics of amelogenesis imperfecta categorized clinically based on the specific type of defect and the disrupted stages of enamel formation: **(A)** hypoplastic; **(B)** hypomature; and **(C)** hypomineralized

Knowledge of the various forms of AI is of fundamental importance in the context of an interdisciplinary treatment concept (Fig. 1). The predominant clinical manifestation in 60–73% of affected individuals is enamel hypoplasia, where properly mineralized tooth enamel is thin or missing (enamel agenesis). Less commonly, enamel hypomineralization occurs, presenting as hypomaturation (20–40%) and hypocalcification (7%), which can occur in isolation or in combination. The clinical picture of AI is characterized by enamel that is more or less soft, dull, and opalescent, ranging from opaque white to honey-colored [8, 11]. Various classifications of AI have emerged since the initial categorization into hypoplastic and hypocalcified types in 1945 [12–19]. Some classifications rely solely on the phenotype (appearance), while others consider the phenotype to be the primary differentiator and incorporate the mode of inheritance as a secondary element in the diagnostic process. Nevertheless, the most common classification is based on categorization according to the predominant phenotype, which is clinically distinguished among four primary groups of AI, as follows [17]:

- Hypoplastic amelogenesis imperfecta (type I) is characterized by a quantitative alteration in enamel thickness, either localized or generalized (Fig. 2A). The teeth display a color ranging from yellow to light brown, with a rough surface texture featuring pits or larger defects. This severe hypoplastic phenotype results in observable morphological irregularities in radiographic examinations. Although typically devoid of pain, occasional reports of mild thermal sensitivity may arise with this type of AI [20].

- Hypomature amelogenesis imperfecta (type II) is distinguished by a deficiency in matrix protein degradation (Fig. 2B). In the enamel, which is the most heavily calcified structure in the body, proper crystal growth necessitates the breakdown and removal of proteins. In type II, the enamel appears white or brown without translucency. Despite normal hardness upon probing and intact enamel layer thickness, enamel breakdown is common. Radiographically, there is a notable reduction in enamel opacity, especially near the enamel–dentin junction. This variant of amelogenesis imperfecta is often considered the mildest and may frequently evade diagnosis [21].
- Hypomineralized amelogenesis imperfecta (type III) represents the most severe manifestation of AI (Fig. 2C). In this condition, there is a notable decrease in enamel mineralization, resulting in discomfort during chewing and brushing. Additionally, individuals may experience gingivitis and periodontal issues, often accompanied by significant dental calculus buildup. Teeth affected by type III AI are highly sensitive to temperature changes and brushing stimuli. The enamel typically exhibits a dark yellow or brown hue. Radiographically, enamel and dentin may appear similarly dense [22, 23].
- Hypoplastic–hypomature amelogenesis imperfecta with taurodontism (type IV), which represents a combination of types I and II. The phenotype is characterized by thin, hard, yellow-brown discolored enamel and reduced tooth crowns with missing interproximal contacts. Attrition-related melt losses play a minor role [24, 25]. The pulp chambers are significantly enlarged in the apico-occlusal direction. The normally shaped tooth crown is supported by a wide, massive root body, which only becomes multi-rooted far apically.

Other key symptoms of AI include hypersensitivity and rapid tooth attrition with a loss of vertical dimension. Gingivitis or gingival hyperplasia is frequently observed as an accompanying feature [11, 26]. The consequences include functional and aesthetic limitations, with many patients reporting disrupted social behavior and psychological issues [27]. AI may co-occur with other dentofacial disorders, such as delayed tooth eruption, missing teeth, denticles, pathological crown and root resorption, and taurodontism [11, 28]. Additionally, there is an increased tendency for impacted permanent teeth and follicular cysts [2]. A frontal open bite can occur depending on the AI type, with a prevalence of 30–60% [29–31].

In 2002, Germany implemented the system of orthodontic indication groups (Kieferorthopädische Indikationsgruppen, KIG) to identify dentofacial disorders warranting treatment (Table 1) [32]. This system, derived from the Index of Orthodontic Treatment Need (IOTN), regulates access to orthodontic care within the German public health insurance framework by evaluating malocclusion severity [32–34]. Craniofacial irregularities such as cleft palate and syndrome-associated oral manifestations are classified as the most severe, while minor contact point displacements, less than 1 mm, indicate the lowest severity level. Notably, malocclusions associated with amelogenesis imperfecta (AI) have not yet been delineated within this system.

**Table 1:** Utilizing the German orthodontic indication groups (KIG), orthodontic treatment need is classified. In the public health insurance system, a severity grade score of 3 or higher acts as the criterion for commencing orthodontic treatment in children below 18 years of age

Malocclusion		Severity grade				
		1	2	3	4	5
A	Craniofacial Anomalies					Cleft palate and syndromes
U	Missing teeth				Agenesis or loss	
S	Disturbance in tooth eruption				Impaction	Displacement
D	Sagittal discrepancy increased overjet	< 3 mm	3–6 mm		> 6–9 mm	> 9 mm
M	Sagittal discrepancy negative overjet				0–3 mm	> 3 mm
O	Vertical discrepancy open bite	< 1 mm	> 1–2 mm	> 2–4 mm	> 4 mm, habitually open	> 4 mm, skeletally open
T	Vertical discrepancy deep bite	> 1–3 mm	> 3 mm, with / without mucosal contact	> 3 mm, with traumatic mucosal impingement		
B	Transverse discrepancy				Scissors bite	
K	Transverse discrepancy crossbite		Buccolingually cusp-to-cusp relation	Bilateral crossbite	Unilateral crossbite	
E	Contact point displacement	< 1 mm	> 1–3 mm	> 3–5 mm	> 5 mm	
P	Space deficiency		< 3 mm	> 3–4 mm	> 4 mm	

**Table 2:** A: Clinical initial situation and orthodontic findings with regard to a study group of 19 patients diagnosed amelogenesis imperfecta

Patient	Gender	AI			Initial findings according to KIG			Anterior teeth		Transversal dental arch difference (Δ)		Tooth anomalies				
		Type	Affected dentition		1°	2°	3°	OJ	OB	anterior	posterior	Missing tooth	Supernumerary tooth	Displacement (D) Retention (R)	Taurodontism	Radiological enamel classification
			1st	2nd												
1	f	2	yes	yes	D4	T2	E2	6.5	4.5	3.1	-2.0	0		none	no	2
2	m	2	NA	yes	S5	D4	T2	6.5	3.5	-2.0	-0.5	0		D (13, 23)	no	1
3	m	1	yes	yes	S4	T2	E2	4.7	6.0	-2	-0.5	1 (26)		R (34)	no	2
4	f	1	NA	yes	E3	P3	D2	6.0	3.0	2.5	-1.7	0		none	no	1
5	m	1	NA	yes	M4	P4	O3	0.0	-3.0	-4.0	-0.9	0		none	no	2
6	f	1	NA	yes	D5	T2	E2	11.5	6.0	-1.6	-0.9	0		none	no	3
7	m	3	yes	yes	M4	K4	O3	0.0	-2.5	-4.0	-5.8	0		none	no	2
8	m	4	NA	yes	U4	T2	E2	2.5	5.0	1.3	-2.8	2 (35. 45)		none	yes	1
9	m	1	yes	yes	S5	B4	O3	2.0	-2.3	5.1	-0.1	0	1 (21)	D (21)	no	3
10	f	1	NA	yes	S4	D2	T2	5.5	4.5	0.5	-2.5	0		R (23)	no	3
11	m	2	yes	yes	M4	K4	O3	0.0	-3.0	-3.7	-3.9	0		none	no	2
12	f	2	yes	yes	E2	D1	T1	2.5	3.8	0.5	-0.5	0		none	no	2
13	f	3	NA	yes	D5	O4	K4	10.0	5.0	-2.5	-8.5	1 (47)		none	no	3
14	m	2	yes	yes	B4	D2	T2	4.5	5.0	5.2	-2.0	0		none	no	2
15	m	1	yes	yes	D4	T2	E2	7.0	6.0	1.0	-2.0	0		none	no	3
16	f	1	yes	yes	S4	K4	T2	1.5	6.0	1.0	-4.0	0		R (27)	no	4
17	f	1	yes	yes	K3	P2	E2	1.5	1.5	-1.5	-4.5	0		none	no	4
18	m	1	yes	yes	S4	O4	K3	1.0	4.5	-2	-1.2	0		R (23)	no	4
19	f	1	yes	yes	O5	S4	E2	2.0	-5.0	-1.5	-4.1	0		none	no	4
Mean, SD								3.96 ± 3.36	-0.02 ± 3.72	-0.91 ± 2.82	-4.39 ± 2.16					

f: female, m: male; OJ: Overjet. OB: Overbite. Δ: Upper – Lower dental arch. SD: Standard deviation; NA: not available

Therefore, the present retrospective study was conducted to identify initial orthodontic findings in patients with AI concerning to the KIG system to determine possible corresponding malocclusions with regard to the AI classification.

## Materials and methods

Approval for conducting this retrospective study was granted by the institutional review board of the Ethics Commission at Witten/Herdecke University, Germany (reference no. S-194/2022). The analysis was conducted on individuals with clinically evident or molecular-genetically diagnosed AI, who sought interdisciplinary treatment at the Department of Orthodontics, Department of Oral Surgery, or Special Care Dentistry at the Witten/Herdecke University between July 2011 and October 2023. For all involved patients the orthodontic diagnostic was performed to evaluate the need for orthodontic treatment in order to plan appropriate orthodontic treatment.

To qualify for participation in the present analysis, potential patients had to be sufficiently compliant to generate orthodontic diagnostic documentation. This included study models, a panoramic radiograph, a lateral cephalogram, as well as clinical intraoral and extraoral photographs. Totally, 19 patients were identified and included in the investigation.

### Clinical analysis and AI classification

The clinical findings were described according to the KIG classifications (Table 1) and the AI classification according to Witkop et al. [17, 32]. They were based on clinical inspections of the oral cavity and dentition as well as evaluations of panoramic radiography and subsequently generated study models.

**Fig. 3:**
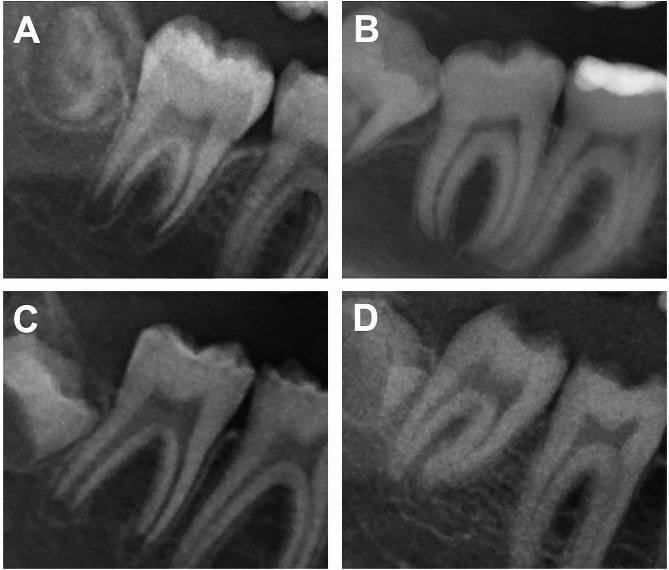
Radiological categorization of the amount of enamel: **(A)** physiological enamel radiodensity; **(B)** reduced enamel radiodensity approximating dentin; **(C)** grossly irregular missing enamel; and **(D)** totally missing enamel

- *Main findings*: Primary, secondary, and tertiary findings of utmost severity were identified from the initial diagnostic records based on the KIG classification in descending severity of the findings (Table 1).
- *Tooth anomalies*: Hypodontia, tooth retention, and displacement in terms of type and number were assessed through panoramic radiography and analysis of study models. Additionally, signs for taurodontism were documented.
- *Model analysis*: Linear measurements included assessments of anterior tooth relation by overjet (mm) and overbite (mm), as well as transversal dental arch relations. The maxillary dental arch width was determined by measuring the distances between both first premolar or primary molar central fissures (P1_Up_/PM1_Up_) as well as between both first molar central fissures (M1_Up_). Similarly, the mandibular dental arch width was determined between both first premolar or primary molar distal marginal ridges (P1_Low_/PM1_Low_) and both first molar distobuccal cusp tips (M1_Low_). The difference (Δ) between the respective distances was calculated to ascertain the relation between maxillary and mandibular arch widths: anterior = P1Up/PM1_Up_ - P1Low/PM1_Low_ and posterior = M1Up - M1Low.
- *Amelogenesis imperfecta expression*: AI was categorized into types I–IV according to Witkop et al. [17]. To determine type IV, a panoramic radiograph was used to identify taurodontism. In addition, the extent of the dentition involved (primary and permanent dentition) as well as a radiologically recognizable amount of enamel were determined from the following: (1) physiological enamel radiodensity; (2) reduced enamel radiodensity approximating to dentin; (3) grossly irregular missing enamel; and (4) totally missing enamel (Fig. 3). Therefore, the first molars were considered, or in cases where restorative measures had already taken place, the adjacent second molars were examined.

### Cephalometric analysis

Sagittal and vertical cranial structures were assessed using established cephalometric measurements [35–37]:

- Sagittal relations: variations (delta, Δ) from ANB (sagittal interbase angle, in degrees) and WITS appraisal (in millimeters).
- Vertical relations: discrepancies (delta, Δ) from NL/NSL (maxillary inclination, in degrees), ML/NSL (mandibular inclination, in degrees), ML/NL (vertical interbasal relationship, in degrees), and the gonial angle (ArGoMe, in degrees).
- Tooth angulations: discrepancies (delta, Δ) from UP1-NSL (upper incisor inclination, in degrees) and LO1-ML (lower incisor inclination, in degrees).

## Results

This study cohort consisted of 19 individuals, with nine female and ten male patients. The mean age at the time of the first appointment was 12.27 ± 4.06 years.

**Fig. 4:**
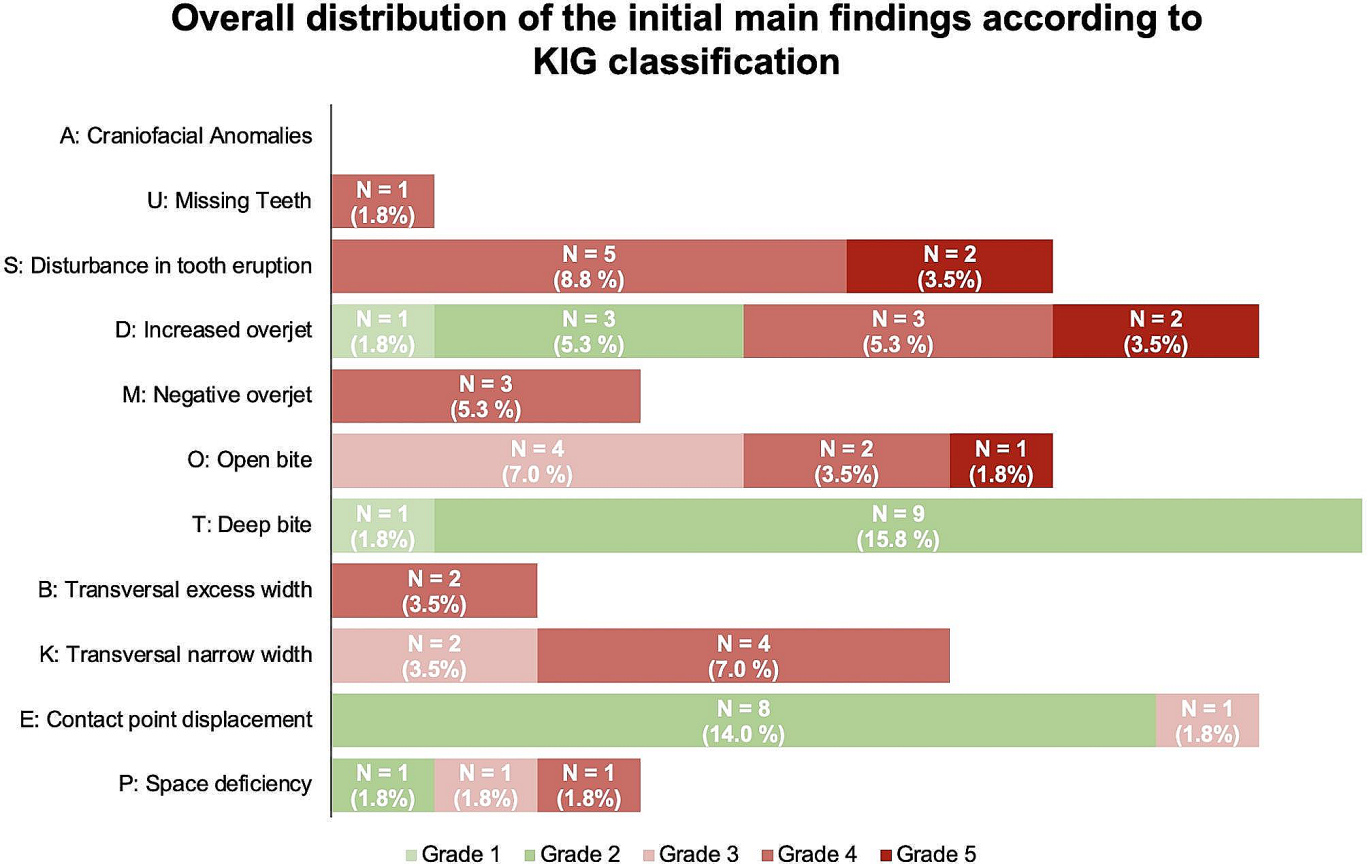
Percentage overall distribution of all initial main findings (100%) within the study group according to the German KIG classification. Green indicates moderate malocclusion, while red indicates severe malocclusions requiring treatment

**Fig. 5:**
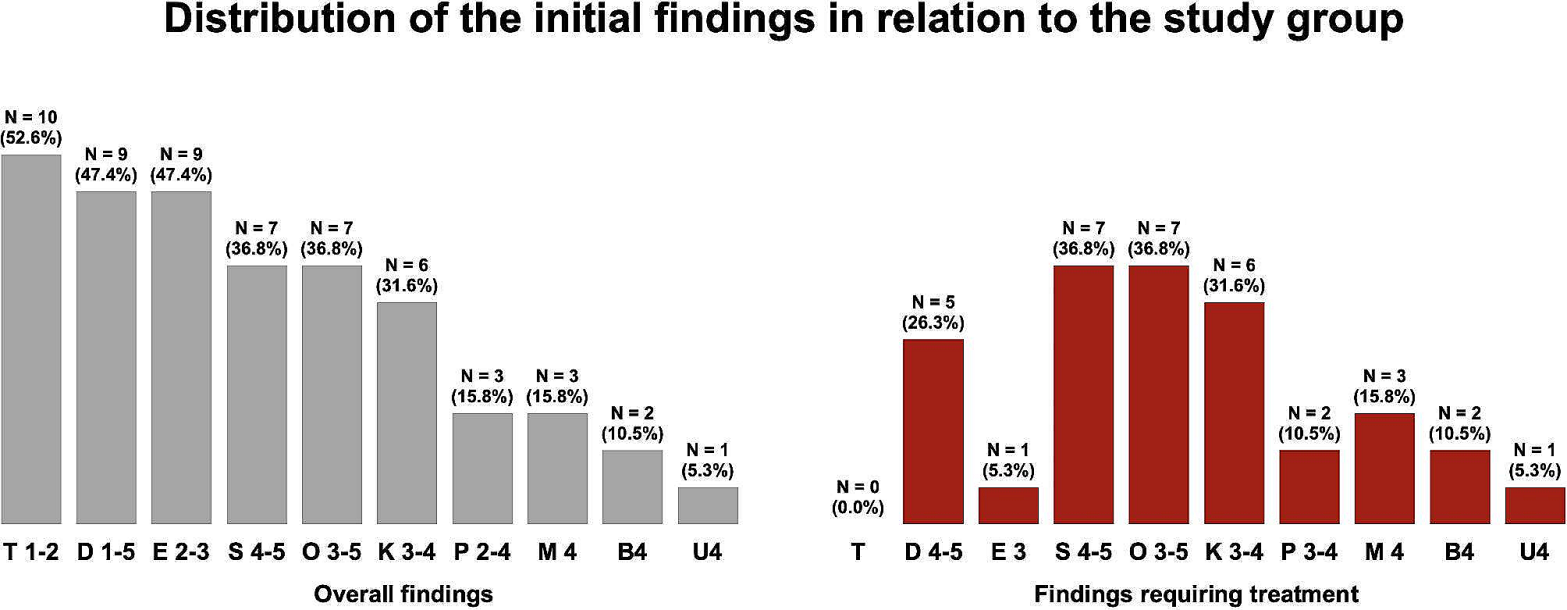
Percentage distribution of individual orthodontic findings in relation to the study group (100%) according to the German KIG classification. Gray indicates moderate malocclusion, while red indicates severe malocclusions with treatment need

Table 2 provides an overview of the initial clinical situations of all 19 patients, while Fig. 4 presents the percentage distribution of the overall initial main findings by the KIG classification concerning the AI type. Approximately 36.8% (*n* = 7) of patients were unable to definitively determine whether the first dentition was affected. In terms of the clinical initial conditions and intermaxillary relationships of the dental arches, an increased overjet (3.96 ± 3.36 mm) and a tendency toward a frontally open bite (-0.02 ± 3.72 mm) were observed. Specifically, the posterior dental arch of the maxilla exhibited a slight narrowing (-4.39 ± 2.16 mm). According to the KIG classification, the most prevalent diagnosis among all cases was a moderate deep bite (T1–T2; 17.6%, *n* = 10), followed by a moderate to severe increased overjet (D1–D5; 15.9%, *n* = 9) and moderate contact point displacement (15.9%, *n* = 9). Only one patient did not require treatment, agreeing to the KIG classification. However, concerning the findings regarding treatment need in relation to the study group, the most common results were disturbances in tooth eruption as well as vertical discrepancies in terms of an open bite (both 36.8%, *n* = 7), as shown in Fig. 5.

Regarding the radiological situation of the patients, Table 3 reveals an approximately even distribution between classes II and III for the majority of the study group. Specifically, eight patients showed class II (42.1%), seven class III (36.8%), and four class I (21.1%). Concerning the vertical dimension, on average, a slight trend toward a dolichofacial skull configuration was found (Δ ML-NSL: 5.07 ± 9.23, Δ ML-NL: 4.24 ± 8.04°). However, only 47.4% (*n* = 9) exhibited a dolichofacial configuration, 36.8% (*n* = 7) a mesofacial configuration, and 15.8% (*n* = 3) a brachyfacial configuration. Concerning the inclination of the front teeth, no significant difference was observed compared to the normal range for the upper and lower anterior teeth (UP1-NSL: -0.42 ± 6.76°; LO1-ML: 2.92 ± 5.55°).

**Table 3:** B: Cephalometry initial findings with regard to the skelettal sagittal and vertical craniofacial configuration of a study group of 19 patients diagnosed amelogenesis imperfecta

Patient	Age	Gender	AI	Sagittal cranial configuration			Vertical cranial configuration					Anterior tooth angulation	
	First Visit			ΔWITS (NR: 0 ± 1 mm)	Δ ANB (NR: 2.0 ± 2°)	Overall Outcome (class)	Δ ML-NSL (NR: 32 ± 2°)	Δ NL-NSL (NR: 8.5 ± 2°)	Δ ML-NL (NR: 23 ± 3°)	Δ ArGoMe (NR:128 ± 7°)	Overall Outcome	Δ UP1-NSL (NR: 102 ± 2°)	Δ LO1-ML (NR: 90 ± 3°)
1	8.6	f	2	3.9	7.3	II	9.3	0.1	9.2	-0.4	dolichofacial	0.7	5.1
2	13.8	m	2	-2.1	-0.8	III	-5.5	2.6	-8.1	-12.2	brachyfacial	2.2	9.3
3	13.3	m	1	0.1	1.4	I	-8.6	-5.9	-2.7	2.9	brachyfacial	10	7.1
4	6.3	f	1	6.1	4.9	II	4.9	8.3	-3.4	2.9	mesofacial	-2.5	-0.7
5	7.7	m	1	-3.3	-5.2	III	6.9	4	2.9	0.4	dolichofacial	-0.4	2.9
6	10.3	f	1	6.5	12.1	II	8.6	3.4	5.2	0.9	dolichofacial	10.2	6.6
7	17.8	m	3	-4.1	-0.3	III	13.7	2.2	11.5	9.5	dolichofacial	1	4.1
8	15.4	m	4	-4.3	0.6	III	-1.2	-1.1	-0.1	-7.6	mesofacial	-9.5	-2.7
9	11.2	m	1	3.7	-6.8	III	7.2	-0.8	8	4.1	dolichofacial	-0.3	-2.9
10	19.5	f	1	3.8	7	II	-7.1	-1.7	-5.4	-4.7	brachyfacial	6.1	8.2
11	8.4	m	2	-4	-0.5	III	11.3	6.8	4.6	11.4	dolichofacial	-4.1	-2.9
12	9.4	f	2	0.6	1.4	I	0.3	1.0	1.0	-1.9	mesofacial	-5.9	2.5
13	20.4	f	3	6.1	9.2	II	27.1	1.1	26	4.7	dolichofacial	12	5.4
14	16.1	m	2	2.4	4.2	II	-2.9	1.1	-4	3.8	mesofacial	3.5	18
15	9.4	m	1	2.3	5.3	II	3.2	5	8.2	4.3	mesofacial	-9.2	2.2
16	10.0	f	1	0.8	0.2	I	-2	-2	0	-12	mesofacial	-6.5	-1.6
17	10.0	f	1	0.9	2.5	I	-0.2	-5.2	5	-2.7	mesofacial	-11.2	-0.2
18	14.1	m	1	-4.7	-1.6	III	8.5	-1	9.5	3.2	dolichofacial	0.6	0
19	11.4	f	1	-0.8	5.7	II	18.1	8.2	9.9	6.9	dolichofacial	-4.6	-5
Mean. SD	12.27 ± 4.06			0.7 ± 3.74	2.45 ± 4.76		5.07 ± 9.23	1.39 ± 4.10	4.24 ± 8.04	0.90 ± 6.57		-0.42 ± 6.76	2.92 ± 5.55

AI: Type of amelogenesis imperfecta NR: Normal range. Δ: Difference to norm. *: Deviation from the norm

.

**Fig. 6:**
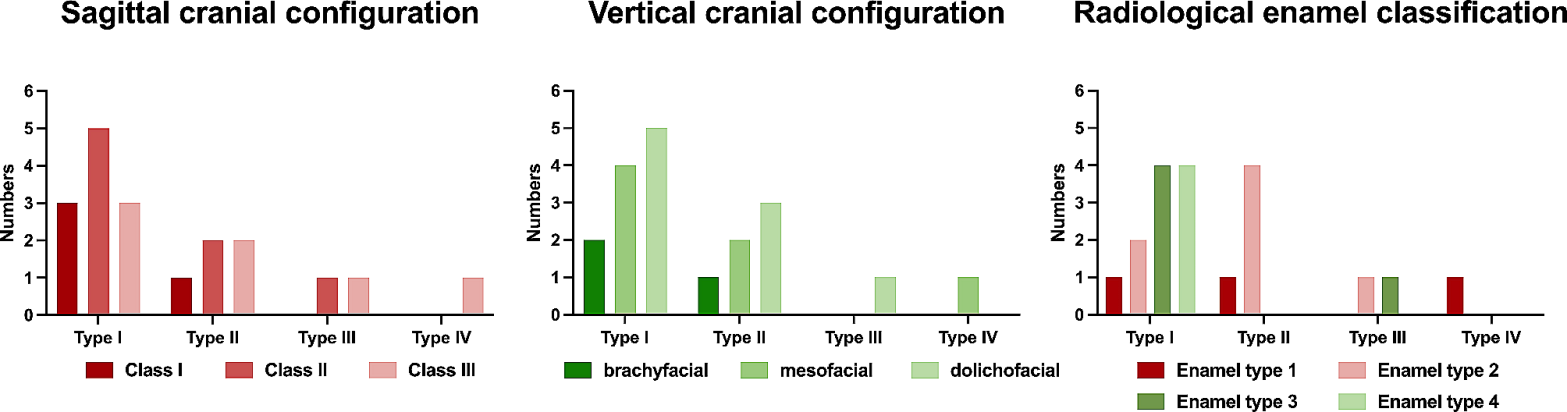
Distribution of the sagittal and vertical cranial configuration as well as radiological enamel classification depending on AI types I–IV.

**Fig. 7:**
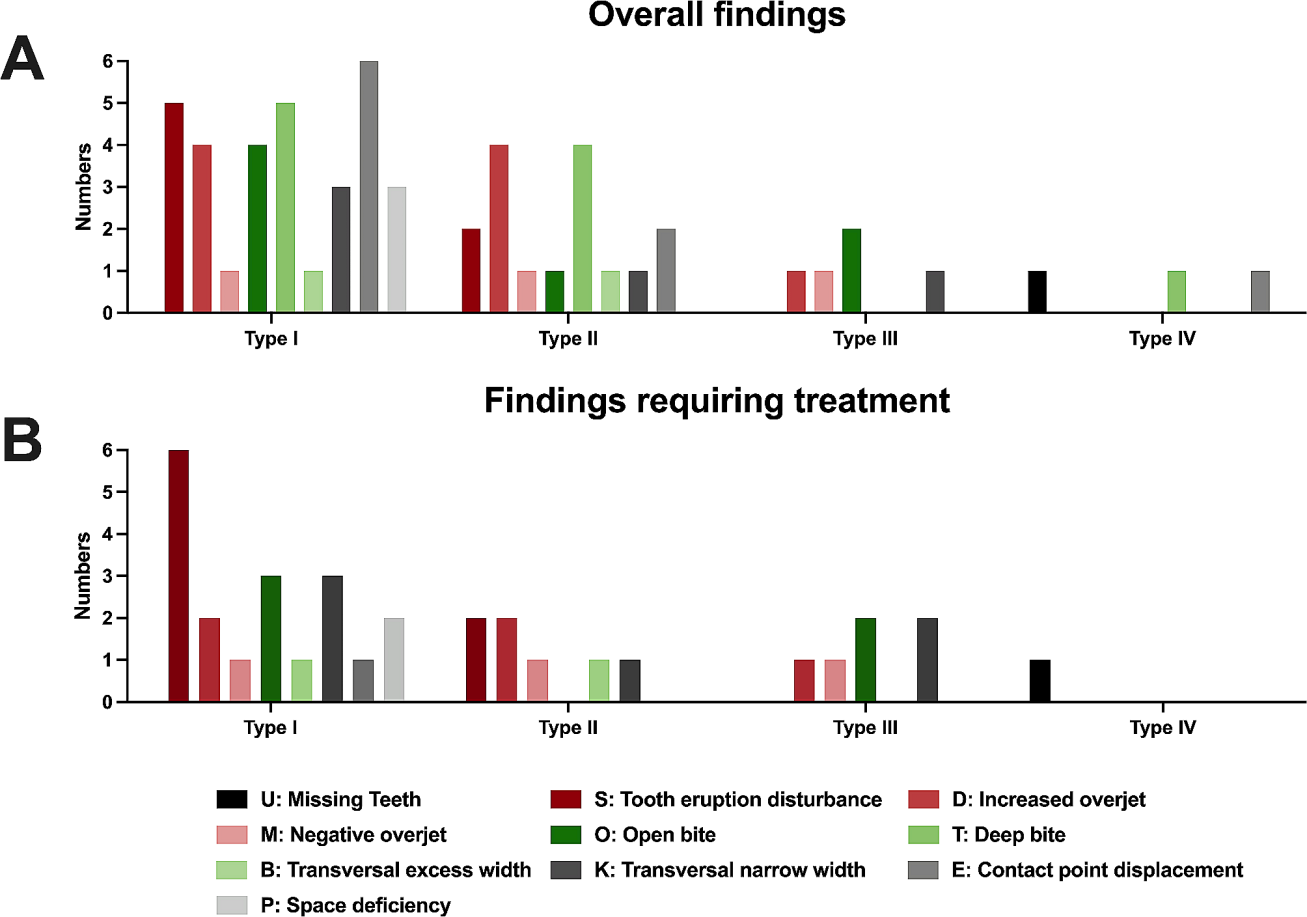
Distribution of the **(A)** overall findings and **(B)** initial findings requiring treatment depending on AI types I–IV.

Figures 6 and 7 depict the distribution of radiological and clinical findings with regard to the AI classes. Concerning the AI type, the overall prevalence was notably higher in individuals with hypoplastic (57.9%, *n* = 11) and hypomature AI (26.2%, *n* = 5), while it was lower in the hypomineralized (10.5%, *n* = 2) and mixed form (5.2%, *n* = 1). With regard the cranial configuration, AI types I and II show an almost even distribution in the sagittal dimension, while the dolichofacial configuration was found most frequently in the vertical dimension. Due to the small number of AI type III and IV cases, no definitive conclusions can be drawn regarding the cranial configuration. With regard to the radiological enamel condition, AI type I cases showed grossly irregular to completely absent enamel (enamel types 3 and 4), while AI type II cases mainly showed reduced enamel radiolucency (enamel type 2). Furthermore, no relationships seem to exist between the AI class and the overall initial main or those requiring treatment.

## Discussion

AI phenotype traditionally focuses on enamel structure alterations, it appears pertinent to broaden the scope to encompass craniofacial aspects, encompassing dento-alveolar or even skeletal malocclusions. Only a few studies have delved into detailed occlusal outcomes in individuals diagnosed with AI so far [31, 38, 39]. In general, occlusal descriptions have often been superficial, primarily noting the presence of an open bite (OB) [40–42], which ranges from 24 to 60% [16, 43–45]. In this context, there appears to be no apparent correlation between the extent of the enamel phenotype and the presence or severity of an anterior open bite [31]. Thus, skeletal open bite can occur across all subtypes of AI, although it is more frequently observed in the hypocalcification type and less so in the hypoplastic type [44, 46]. Already Rowley et al. reported that cases of hypomaturation-type AI completely lack a manifestation of a skeletal open bite [44]. In a recent systematic review exploring the association between malocclusion and genotype and phenotype of AI, Broutin et al. observed that all AI phenotypes exhibited an open bite rate of approximately 35%, except for the mixed form [47]. Other malocclusions were rarely discussed, and a clear correlation between occlusal phenotype and genotype or AI phenotype was also not clearly described, which was attributed to the limited occlusal descriptions and small sample sizes of patients. It was concluded that, up to now, the documentation of open bite malocclusions has been more common in the context of AI [47]. Therefore, a more precise delineation of the orofacial features associated with AI is needed.

In this study, we conducted a retrospective investigation of patient data with AI to identify those for whom a treatment indication had been determined. On average, the patients were 12.27 ± 4.06 years old, which aligns with the age range typically seen in orthodontic patients undergoing university orthodontic therapy, approximately 12.1 ± 3.5 years [48]. The degree of malocclusion was assessed using the German KIG classification and involved an assessment of the initial clinical and radiological conditions. KIG closely resembles the IOTN in terms of group structure and severity grade; but it does not consider subjective aesthetic assessments [32, 49]. Until now, it has not been described to classify patients with AI [32]. This assessment enabled identification of malocclusions in transversal dimension as well as tooth-specific findings such as missing, retained, and displaced teeth. In the current investigation, the primary observations requiring treatment encompassed malocclusions linked to an open bite (O3–O5; *n* = 7, 36.8%), but also disruptions in tooth eruption (S4–S5; *n* = 7, 36.8%) characterized by retention or displacement as well as lateral edge-to-edge or cross bites (K3–K4; *n* = 6, 31.6%) and sagittal discrepancies marked by an increased overjet (D3–D5; *n* = 5, 26.2%).

Concerning the skeletal configurations findings, an approximately equal distribution between classes II (*n* = 8, 42.1%) and III (*n* = 7, 36.8%) was found, why is not clearly reflected in terms of the average variance in results for the skeletal configurations, which means that an ANB is about 2.45 ± 4.76° and a WITS value is about 0.7 ± 3.74 mm, on average. However, a slight inclination toward a dolichofacial configuration (ΔML-NSL: 5.07 ± 9.23°, ΔML-NL: 4.24 ± 8.04°). Thus, the results seem to be in line with the current literature. For example, Messaoudi et al. recently reported in a meta-analysis on craniofacial cephalometric characteristics that subjects with AI demonstrated a larger ANB angle (SMD = 0.61; 95% CI 0.34, 0.89; *p* < 0.01) than those of the control groups in the sagittal axis [50]. Furthermore, in the vertical axis, those with AI presented a smaller overbite (SMD = − 1.15; 95% CI − 2.22, − 0.08; *p* = 0.04) and larger intermaxillary angle (SMD = − 1.15; 95% CI − 2.22, − 0.08; *p* = 0.04) than those without AI.

In terms of the current distribution of AI types, the highest proportion was found for hypoplastic (57.9%, *n* = 11) and hypomature AI (26.2%, *n* = 5), while lower proportions were discovered for hypomineralized (10.5%, *n* = 2) and mixed types (5.2%, *n* = 1). Unfortunately, no relationship seems to exist between the four different AI phenotypes and the radiological imaging of the enamel structure in the panoramic radiograph. This could be due to the fact that the pitted or striae enamel of hypoplastic form, for example, could not be clearly differentiated radiologically from intact enamel in terms of radiopacity. In addition, early enamel loss can also radiologically mimic hypoplasia of the aplasia. Although it appears that reduced radiopacity or aplasia of the enamel may indicate AI, physiological radiographic density cannot lead to the exclusion of AI. Additionally, no association between open bite malocclusion and a specific AI phenotype appears to be present. This lack of association also extends to the findings regarding sagittal and vertical skull configuration across different AI types. Additionally, there no relationship between the AI phenotype and the overall main findings, and those findings with a real treatment need appears to be present. This is probably due to the small number of cases. In this context, Broutin et al. described in a systematic review the association between malocclusions and the genotype and phenotype of AI [47]. According to their findings, open bite emerged as the most frequently observed malocclusion, accounting for 27.7% (*n* = 94) of cases, irrespective of the enamel phenotype as per Witkop’s classification. Concerning the AI type, the prevalence was notably higher in individuals with hypoplastic (35.5%, *n* = 38) and hypomineralized AI (36.7%, *n* = 25), while it was lower in mixed forms (9.8%, *n* = 9) [47]. Furthermore, cephalometric radiograph studies assessing occlusion revealed that open bite occurred in 46.8% of individuals with hypoplastic (*n* = 22) and hypomatured AI (*n* = 15) as well as in 45% of those with the hypomineralized AI phenotype (*n* = 18), but not in mixed forms. Posterior open bite was more prevalent in the hypoplastic form of AI. Class I in Angle’s classification was the most commonly observed, although some data were not available.

There are some limitations that it is critical to consider regarding the evaluation and interpretation of the present data. On one hand, there is generally a low incidence rate with variations across distinct demographic groups, ranging from 1:14,000–16,000 to 1.4:1,000 [51–53]. This low incidence is also reflected in the number of cases in the present investigation. In addition, the clinical presentation of AI encompasses a broad spectrum of phenotypes typified by hypomineralization and/or hypoplasia coupled with discolouration, sensitivity, and enamel breakability. There are also differences in the frequency of the four primary AI phenotypes outlined by Witkop et al. [17]. Thus, the predominant clinical manifestation is enamel hypoplasia (60–73%), followed by hypomaturation (20–40%) and hypocalcification (7%). The present patient group also showed a similar distribution pattern. This made an AI type-specific classification of orofacial findings difficult and limited the meaningfulness of the available results. In this context, it should also be critically noted that classification according to the four main AI groups was based on clinical findings and the exclusion of other dental hard tissue anomalies, since genetic confirmation is only currently available in a few cases. Finally, it must also be critically considered with regard to the initial orthodontic findings that the patients presented at different times during tooth replacement, which is very likely to have an effect on the clinical and radiological outcome.

Nevertheless, the present data indicate for the first time that in addition to the already known increased appearance of anterior open bite, there may also be a higher incidence of disruptions in tooth eruption as well as transversal discrepancy of the maxillary dental arch. In the long-term, enhanced understanding of the risk of AI-associated dysgnathia, whether based on phenotype or genotype, should enable these findings concerning the management of orofacial dysfunction and teeth eruption to be addressed. In this context, the risk of uncertain bonding failure of orthodontic braces with regard to corresponding enamel abnormalities could be positively affected.

## Conclusion

AI is not clearly associated with possible clinical and radiological orthodontic findings. In addition to the known tendency toward an open bite, there also appears to be a higher prevalence of disruptions in tooth eruption and laterally edge-to-edge or cross bites. However, both the present results and those of previous studies show no association with the four main AI types. Regarding the significant expression levels of genotype and phenotype, additional investigations into gene-specific relationships could provide valuable clarification.

## Data Availability

No datasets were generated or analysed during the current study.
